# Cortical Surface Area Correlates with *STON2* Gene Ser307Pro Polymorphism in First-Episode Treatment-Naïve Patients with Schizophrenia

**DOI:** 10.1371/journal.pone.0064090

**Published:** 2013-06-13

**Authors:** Bo Xiang, Jun-yao Wu, Qiang Wang, Ming-Li Li, Li-Jun Jiang, Wei Deng, Zhuang-Fei Chen, Zong-Ling He, Cao-Hua Huang, Yuan-yuan Han, Yin-fei Li, Yin Lin, Xiang Liu, Ying-cheng Wang, Xiao-Hong Ma, Qi-yong Gong, Tao Li, Xun Hu

**Affiliations:** 1 State Key Laboratory of Biotherapy, West China Hospital, Sichuan University, Chengdu, Sichuan, China; 2 The Mental Health Center and the Psychiatric Laboratory, West China Hospital, Sichuan University, Chengdu, Sichuan, China; 3 Huaxi MR Research Center, Department of Radiology, West China Hospital, Sichuan University, Chengdu, Sichuan, China; 4 Huaxi Biobank, West China Hospital, Sichuan University, Chengdu, Sichuan, China; Rikagaku Kenkyūsho Brain Science Institute, Japan

## Abstract

**Background:**

Evidence shows that *STON2* gene is associated with synaptic function and schizophrenia. This study aims to explore the relationship between two functional polymorphisms (Ser307Pro and Ala851Ser) of *STON2* gene and the cortical surface area in first-episode treatment-naïve patients with schizophrenia and healthy controls.

**Methodology/Principal Findings:**

Magnetic resonance imaging of the whole cortical surface area, which was computed by an automated surface-based technique (FreeSurfer), was obtained from 74 first-episode treatment-naïve patients with schizophrenia and 55 healthy controls. Multiple regression analysis was performed to investigate the effect of genotype subgroups on the cortical surface area. A significant genotype-by-diagnosis effect on the cortical surface area was observed. Pro-allele carriers of Ser307Pro polymorphism had larger right inferior temporal surface area than Ser/Ser carriers in the patients with schizophrenia; however, no significant difference was found in the same area in the healthy controls. The Ala851Ser polymorphism of *STON2* gene was not significantly associated with the cortical surface area in patients with schizophrenia and healthy controls.

**Conclusions/Significance:**

The present study demonstrated that the functional variant of the *STON2* gene could alter cortical surface area on the right inferior temporal and contribute to the pathogenesis of schizophrenia.

## Introduction

Schizophrenia is a complex psychiatric disorder characterized by various clinical symptoms, including delusions, hallucinations, and cognitive impairments. Studies suggested that genetic factors influence the pathophysiology of schizophrenia with heritability as high as 80% [1], [2]. During the last two decades, association analysis results, including recent genome-wide association studies, have provided evidence for a substantial polygenic component to the risk of schizophrenia, with a minimal effective size [3], [4], [5], [6], [7]. Despite numerous findings mainly based on statistical evidence, the genetic mechanisms in schizophrenia remain largely unknown [8]. Potential factors such as allelic heterogeneity, variation in population substructure, and phenotypic heterogeneity have hindered progress in this field. Considerable interest has been demonstrated in reducing the effect of phenotypic heterogeneity by integrating endophenotypes such as neurocognitive deficits or neuroimaging-based phenotypes into molecular genetic analysis [9], [10], [11], [12].

Studies showed that some gene expression domains, as well as several transcription factors and signaling molecules, were involved in establishing area boundaries in the mouse neocortex. These findings suggested that genetic factors remarkably influenced the regional differentiation of the brain [13], [14], [15], [16]. This study has showed that genetic patterning based on the cortical divisions of cortical surface area and anteroposterior gradient was found in mouse models and human cortex [17], and the expansion of region-specific cortical areal in humans was involved with specific genetic polymorphisms [18], [19]. Chen et al. recently reported that the human cortical surface area was a hierarchical genetic organization [20]. At birth, surface area is influenced by the number of cortical columns and cortical thickness, and which continue to grow until ∼8 to 10 years after birth [21], [22]. Thus for, the human cortical surface area may be entirely related with genetic, rather than a priori functional or structural information, and cortical surface area may represent potential endophenotype of psychiatric disorders in investigating the potential association of genetic variants and brain morphology as well as in providing novel insights into the pathophysiology of schizophrenia and other related disorders.

The *STON2* gene is located on chromosome 14q, which is a candidate region implicated in linkage studies of schizophrenia [23], [24]. The *STON2* gene encodes a human homolog of Drosophila stoned B–stonin2. Studies demonstrated that *stoned B* inactivation in Drosophila resulted in the inability to reform synaptic vesicles (SVs) after exocytosis [25], [26], [27], [28], [29]. Transport, fusion, and recycling of SV affected the normal synaptic function and were critical in maintaining the ability of the synapse to release a neurotransmitter based on sustained stimulation [30], [31], [32]. Two exonic single-nucleotide polymorphisms (SNPs) of the *STON2* gene were associated with schizophrenia in a Chinese population [33]. The present study aimed to investigate the relationship between the genetic of the *STON2* gene and the cortical surface area in patients with schizophrenia and healthy controls. We hypothesized that functional genetic variants of *STON2* gene, Ser307Pro and Ala851Ser, was associated with the cortical surface area of patients with schizophrenia, and could provide evidence that the *STON2* gene is related to the development of schizophrenia.

## Materials and Methods

### Samples

A total of 129 subjects were recruited, including 74 first-episode, treatment-naïve patients with schizophrenia and 55 healthy controls. All patients were recruited from the Mental Health Centre of the West China Hospital, Sichuan University, PR China. These patients were assessed by trained psychiatrists according to the Structured Clinical Interview for Diagnostic and Statistical Manual of Mental Disorders, Fourth Edition (DSM-IV) Axis I Disorders [34]. The healthy controls were recruited from the local area by advertisement and were screened for a lifetime absence of psychiatric illnesses by using the Structured Clinical Interview for DSM-IV-TR Axis I Disorders-Non Patient Edition [35]. Subjects with significant physical illnesses, pregnancies, or psychiatric disorders other than schizophrenia were excluded. All controls were interviewed to assure that no first-degree relatives had a history of psychiatric illness. The study was approved by the Ethics Committee of the West China Hospital of Sichuan University. All next of kin, carer takers or guardians consented on the behalf of participants to provided written informed consent for their participation.

### Imaging

Data acquisition: A total of 129 participants underwent magnetic resonance imaging (MRI) scans in the Department of Radiology at West China Hospital with a 3 Tesla MRI system (EXCITE, General Electric, Milwaukee, USA) with an eight-channel phased-array head coil. High-resolution T1 images were obtained by three-dimensional spoiled gradient echo sequence from all participants. The sets used in this protocol included the following: TR = 8.5 ms; TE = 3.93 ms; dip angle = 12°; thickness of slice = 1 mm; single shot; field of view = 24 cm×24 cm; matrix = 256×256; size of vowel = 0.47×0.47×1 mm^3^. A total of 156 slices of axial images were collected from a brain. All scans were inspected for motion artifacts, and the absence of gross pathological findings was confirmed by a neuroradiologist.

### MRIs: preprocessing

We used the FreeSurfer software (http://surfer.nmr.mgh.harvard.edu/fswiki), which includes a set of automated tools, to reconstruct the brain cortical surface from the T1-weighted MRIs [36]. This method involved intensity normalization and used a skull-stripping algorithm to remove the extracerebral tissues as well as a connected component algorithm for image segmentation. The output at this stage consists of a single-filled white matter to estimate the gray–white matter interface. The gray–white matter assessment was used as the starting point of a deformable surface algorithm to examine the pial surface. The surface area was obtained according to the shortest distance between equivalent vertices in the pail and gray–white matter surfaces [37] by using a Gaussian smoothing kernel with a full width at half maximum of 10 mm to smooth the surface.

### Genotyping

DNAs were obtained using a standard phenol–chloroform isolation method from whole blood [38]. Ser307Pro and Ala851Ser polymorphisms in *STON2* gene were genotyped by GoldenGate genotyping assay according to the manufacturer's instructions (Illumina Beadstation 500; Illumine, San Diego). All genotypes were tested according to the following quality control criteria: SNPs were removed if more than 10% genotypes across samples were missing, minor allele frequency of SNP < 5%, and SNPs failed the Hardy–Weinberg equilibrium test in healthy controls (i.e., p value <10^−5^) by PLINK [39].

### Statistical analyses

We performed intergroup averaging and inference on the cortical surface data generated by the FreeSurfer processing stream. Patients with schizophrenia and healthy controls were divided into subgroups according to SNP genotypes, respectively: 2× Ser/Ser, 2× Ser/Pro and Pro/Pro (Pro-allele carriers) for Ser307Pro polymorphism; and 2× Ala/Ala, 2× Ser/Ala and Ser/Ser (Ser-allele carriers) for Ala851Ser polymorphism. A general linear model was used to explore the differences in the cortical surface area between the subgroups divided according to genotypes of above two polymorphisms separately in patients at each vertex of the surface, with age and sex as covariance. We also investigated the relationship between cortical surface and PANSS score, and age and sex as covariance. For comparison among the subgroups, the results in the group mapping analysis were saved to a specific file and imported into the label. The mean region of interest (ROI) values were extracted for subsequent calculation for each subject. These values were further analyzed using SPSS version 13.0 for Windows (SPSS Inc., USA). The right and left hemispheres were tested separately. We also used the Monte Carlo cluster wise multiple correction with p<0.05 across the whole brain to reduce the possibility of obtaining false positives. The χ^2^ test and t-test were used to compare the sex, age, and educational attainment years between patients with schizophrenia and healthy controls.

## Results

The demographic characteristics of the sample are summarized in Table 1. No significant difference was indicated in age, sex, and educational years between patients with schizophrenia and healthy controls.

**Table 1:** Demographic and Clinical Data.

Variables	Schizophrenic patients (N = 74)	Healthy controls (N = 55)	p value
Age (years)	25 (8.3)	25 (8.8)	0.981
Sex (male/female)	30/44	25/30	0.094
Education attainment (years)	12.7 (2.9)	12.4 (3.0)	0.622
PANSS-P	24.26 (6.59)		
PANSS-N	18.7 (7.7)		
PANSS-G	47.6 (9.7)		
PANSS-T	88.94 (17.19)		

Values of all variables are mean (S.D.) except sex.

PANSS, Positive and Negative Syndrome Scale; PANSS-P, subscales for positive symptoms; PANSS-N, subscales for negative symptoms; PANSS-G, subscales for general psychopathological symptoms; PANSS-T, total score of PANSS.

Ser307Pro and Ala851Ser polymorphisms of the *STON2* gene did not deviate from Hardy–Weinberg expectations in healthy controls (p = 0.295 and 0.45, respectively). The genotypic and allelic distributions of both polymorphisms are presented in Table 2. No significant difference was indicated in the frequencies of polymorphisms, both genotype-wise and allele-wise, between patients with schizophrenia and healthy controls.

**Table 2:** Genotype distributions and allele frequencies of Ser307Pro and Ala851Ser polymorphisms of STON2 gene among patients with schizophrenia and healthy controls.

	Genotypes			Allele frequency	
Ser307Pro	Ser/Ser	Ser/Pro	Pro/Pro	Ser	Pro
Patients	16 (0.22)	40 (0.54)	18 (0.24)	72 (0.49)	76 (0.51)
Controls	17 (0.31)	30 (0.55)	8 (0.14)	64 (0.58)	46 (0.41)
Ala851Ser	Ala/Ala	Ser/Ala	Ser/Ser	Ala	Ser
Patients	28 (0.38)	38 (0.51)	8 (0.11)	94 (0.635)	54 (0.365)
Controls	26 (0.47)	23 (0.42)	6 (0.11)	75 (0.68)	35 (0.32)

**Figure 1:**
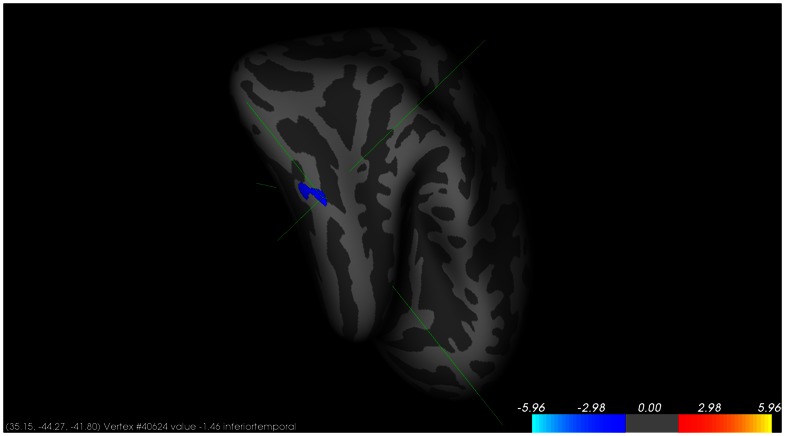
The statistical map of Cortical indicating the significant difference between Ser/Ser and Pro-allele carriers on the right inferior temporal cortical surface area in schizophrenic patients. The labeled cluster represents the right hemisphere region that survived Monte Carlo clusterwise correction at p<0.05. Color bar scaled in negative log of p values.

**Figure 2:**
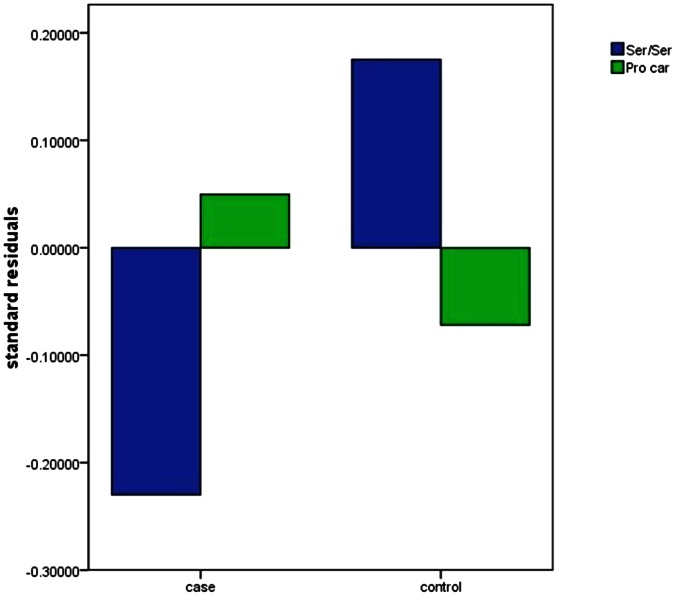
Standard residuals were obtained from an average effect of the right inferior temporal cortical surface area regressing out the effects of sex and age.

No significant difference was indicated in the cortical surface area between patients with schizophrenia and healthy controls. We found a significant difference between Ser/Ser and Pro-allele carriers in the right inferior temporal cortical surface area in patients with schizophrenia (P<0.05 after correction for multiple comparisons across the whole brain) (Figure 1). The mean cortical surface area values obtained from all participants were then extracted in the right inferior temporal cortical surface area. We found that the Ser/Ser carriers with schizophrenia had a significantly smaller cortical surface area on the right inferior temporal hemisphere compared with the Pro-allele carriers with schizophrenia. However, no significant difference was indicated in the same area in healthy controls (Table 3; Figure 2). No association between Ala851Ser and the cortical surface area observed in patients with schizophrenia and healthy controls. There also have no correlation between the cortical surface and the PANSS score.

**Table 3:** Right inferior temporal surface area by groups of patients and controls and by genotypes of Ser307Pro polymorphism in STON2 gene.

Subgroups by genotypes	Ser/Ser	Pro car*	F**	p value
Patients (mm^2^ ± SD)	166.1±39.3	174.1±34.6	3.256	0.027
Controls (mm^2^ ± SD)	201.7±41.1	189.4±36.9	0.830	0.483

* Pro car indicates Ser/Pro and Pro/Pro.

** Sex and age were included as covariates.

## Discussion

In this study, we used the imaging genetics approach to examine the effects of Ser307Pro and Ala851Ser polymorphisms of the *STON2* gene on the cortical surface area in patients with schizophrenia and healthy controls. Our results showed that the Pro-allele carriers of Ser307Pro polymorphism have significant increased right inferior temporal cortical surface area in patients with schizophrenia only.

Schizophrenia has been hypothesized to exhibit an abnormal neurodevelopmental process, which results in reduced brain lobe surface area (e.g., left temporal lobe) [40], [41] and abnormal asymmetry patterns of cortical and subcortical structures [42], [43]. This altered brain lobe surface area and asymmetry patterns in schizophrenic patients may be an endophenotype related to schizophrenia.

In the present study, we identified the occurrence of group-by-genotype interactions in the cortical surface area. However, the mechanism by which the *STON2* Ser307Pro variant affects the cortical surface area remained unclear. Stonin2 is the first endocytic protein, which dedicates specifically to SV recycling by acting as a sorting adaptor for synaptotagmin-1 and serves as a link between the endocytic proteins AP-2 and Eps15 and the calcium-sensing SV protein synaptotagmin 1 [44]. This protein facilitates synaptotagmin-1 redistribution into SVs in primary neurons [45], [46]. The clathrin-mediated endocytosis may be the major mechanism for recycling of fully fused SVs, as well as a major mechanism of dopaminergic signaling attenuation [47], [48]. Studies suggested that dopamine 2 receptors D2 (D2Rs), which plays an important role in the dopaminergic system, are related to the N-methyl-D-aspartate receptor (NMDAR) [49], [50]. NMDAR is essential for synaptic plasticity [51]. Thus, stonin2 may be involved in regulating the internalization of D2R and NMDAR. Some studies

Luan et al. found the positive association of Ser307Pro and Ala851Ser polymorphisms with schizophrenia in a Chinese population. Pro307Ser polymorphism is adjacent to an Asn-Pro-Phe or NPF motif, which may mediate the interaction of stonin2 with intersectin and Eps15, as well as the haplotype C-C of Ser307Pro and Ala851Ser polymorphisms (Pro307-Ala851), which affects the stonin2 function that mediates the etiopathogenesis of schizophrenia [33]. In the present study, no significant difference was found in the frequencies of both Ser307Pro and Ala851Ser polymorphisms in *STON2* gene, either genotype-wise or allele-wise, between patients with schizophrenia and healthy controls. However, we identified that the Ser307Pro variant significantly affected the right inferior temporal cortical surface area in schizophrenic patients, and Anand A et al. has found that the smaller deficits in right inferior temporal cortices in nonpsychotic siblings of patients with childhood-onset schizophrenia [52], Cabeza R et al. found the inferior temporal gyrus are involved in several cognitive processes [53] (such as visual perception [54], [55]) and the functional deficit in this cognitive domain has been reported in schizophrenia [56]. Previous study has found Stonin2 was interacted with synaptotagmin 1 which was greater association with large projection neurons to participate in synaptic vesicle recycling, and projection neurons which were related with cortical expression [44], [57]. So this finding partially supports the positive association of the human *STON2* gene with schizophrenia, as indicated in the study by Luan.

In summary, our study provided preliminary evidence that the functional variant of the *STON2* gene altered the right inferior temporal cortical surface area and contributed to the pathogenesis of schizophrenia. However, the function of *STON2* Ser307Pro remains to be further explored. The mechanism by which the variant influences SV recycling and relates to mental disorders such as schizophrenia must be investigated in the future.
